# Multi-Layered TiO_2_ Films towards Enhancement of *Escherichia coli* Inactivation

**DOI:** 10.3390/ma9100808

**Published:** 2016-09-30

**Authors:** Sorachon Yoriya, Angkana Chumphu, Pusit Pookmanee, Wreerat Laithong, Sirichai Thepa, Roongrojana Songprakorp

**Affiliations:** 1 National Metal and Materials Technology Center, 114 Thailand Science Park, Phahonyothin Road, Khlong 1, Khlong Luang, PathumThani 12120, Thailand; angkanac@mtec.or.th; 2 Department of Chemistry, Faculty of Science, Maejo University, Chiang Mai 50290, Thailand; pusit@mju.ac.th; 3 Energy Technology Division, School of Energy Environment and Materials, King Mongkut’s University of Technology Thonburi, 126 Pracha Uthit Road, Thung Khru, Bangkok 10140, Thailand; wreerat@yahoo.com (W.L.); sirichai.the@kmutt.ac.th (S.T.); roongrojana.son@kmutt.ac.th (R.S.)

**Keywords:** TiO_2_, solar radiation, inactivation, *E. coli*, photocatalytic process

## Abstract

Crystalline TiO_2_ has shown its great photocatalytic properties in bacterial inactivation. This work presents a design fabrication of low-cost, layered TiO_2_ films assembled reactors and a study of their performance for a better understanding to elucidate the photocatalytic effect on inactivation of *E. coli* in water. The ability to reduce the number of bacteria in water samples for the layered TiO_2_ composing reactors has been investigated as a function of time, while varying the parameters of light sources, initial concentration of bacteria, and ratios of TiO_2_ film area and volume of water. Herein, the layered TiO_2_ films have been fabricated on the glass plates by thermal spray coating prior to screen printing, allowing a good adhesion of the films. Surface topology and crystallographic phase of TiO_2_ for the screen-printed active layer have been characterized, resulting in the ratio of anatase:rutile being 80:20. Under exposure to sunlight and a given condition employed in this study, the optimized film area:water volume of 1:2.62 has shown a significant ability to reduce the *E. coli* cells in water samples. The ratio of surface area of photocatalytic active base to volume of water medium is believed to play a predominant role facilitating the cells inactivation. The kinetic rate of inactivation and its behavior are also described in terms of adsorption of reaction species at different contact times.

## 1. Introduction

*Escherichia coli* bacteria found in the contaminated wastewater is known to significantly affect human health. Usually, *E. coli* inactivation can be done by a variety of methods, such as boiling, solar heating, radiating, filtering with filter paper or sheet, and applying certain chemicals to annihilate the cells of microorganisms [1,2,3,4,5]. Solar irradiation is an effectively convenient method of inactivation. Conroy et al. [6] used batch processing of solar inactivation for improving the quality of biologically contaminated drinking water in developing countries. The technique involved storing the contaminated drinking water in the transparent containers, e.g., plastic bags, plastic bottles, and glass bottles that were placed directly under sunlight for eight hours before consumption. McGuigan et al. [7] has also shown the high effectiveness of irradiation on cell inactivation against a broad range of bacterial pathogens.

Titanium dioxide (TiO_2_) is known as an extensively used material offering great potential for photocatalytic inactivation in water [8,9,10,11] and treatment of organic contaminants [12,13,14]. Likewise, Nesic et al. found that the *E. coli* inactivation of TiO_2_ aggregates in the absence of light [15]. A TiO_2_ photocatalyst is used to combine with solar irradiation in order to enhance inactivation of bacteria [16,17,18,19]; the use of commercial TiO_2_ in *E. coli* inactivation has been widely reported in literature [20,21,22]. In the photocatalytic process, TiO_2_ is known to generate the reactive oxygen species such as superoxide radical anion (O_2_^•−^) and hydroxyl radical (HO^•^) [23,24] after the generated electron–hole pairs reacted, respectively, with O_2_ and H_2_O [25]. The hydroxyl radical species is produced when TiO_2_ is excited by UV radiation of wavelength near 390 nm [26]. After being exposed to UV light higher than its band gap energy, the anatase form releases the radical species, resulting in oxidative stresses towards microorganisms and thus cell death [27,28,29,30].

The most active crystalline structure of TiO_2_ for generating free radical species is anatase. Zuccheri et al. reported the use of TiO_2_ nanoparticles in the formulation of interior paints possessing anti-bacterial activity, as the suitable crystalline structure of TiO_2_ in the formulation was found to contain 85% of anatase. Duffy et al. [31] has confirmed that the TiO_2_ coatings can be used to accelerate the inactivation rate of bacterial pathogens, with the addition of TiO_2_ coated inserts resulting in the improved efficiency of bacterial inactivation by 20%–25%. Recently, the Kiwi group has sputtered TiO_2_ on polyethylene (PE) fabrics for antibacterial purpose. The work has revealed the necessity of high-anatase TiO_2_ to induce *E. coli* inactivation in a minute range under simulated sunlight irradiation, with the higher TiO_2_ loading leading to faster bacterial inactivation kinetics on the PE surface [32]. For dye degradation purposes, the mixed TiO_2_ phases with high anatase in the PE-TiO_2_ sputtered film has been found effective in the discoloration of methylene blue (MB) under solar simulated light [33].

Thermal spraying is a feasible and rapid technique to prepare the coating film on plates from the conventional TiO_2_ powder [34], allowing for the titania coatings with enhanced mechanical performance. Nararom et al. [35] reported the inactivation of *E. coli* in water by using solar heating with TiO_2_ inserted plates, fabricated by thermal spray coating, with an aim to study the photocatalytic process associated with a compound parabolic concentrator by solar heating. The reactor surface area of 0.014 m^2^ illuminated by the incoming photon with accumulated solar energy of 10 kJ/L was found sufficient to reduce the number of *E. coli* by two logs colony-forming unit (CFU). However, the continuous flow system of water through the reactor has no significant effect on the improvement of solar inactivation [35].

This work presents a design process of low-cost, layered TiO_2_ films assembled reactor combining with solar water heating and a study of their performance towards the enhancement of *E. coli* inactivation. The fabrication of double-type TiO_2_ film layers consists of thermal spray coating and screen printing layers. To investigate the bactericidal activity of the TiO_2_ films’ assembled reactors, the effectiveness on the cells’ inactivation is compared in view of different light sources, initial cell concentration, and ratio of the fabricated film area per volume of water. The kinetic behavior of the *E. coli* inactivation is elucidated for a better understanding by considering the time dependence of *E. coli* inactivation.

## 2. Results

**Figure 1:**
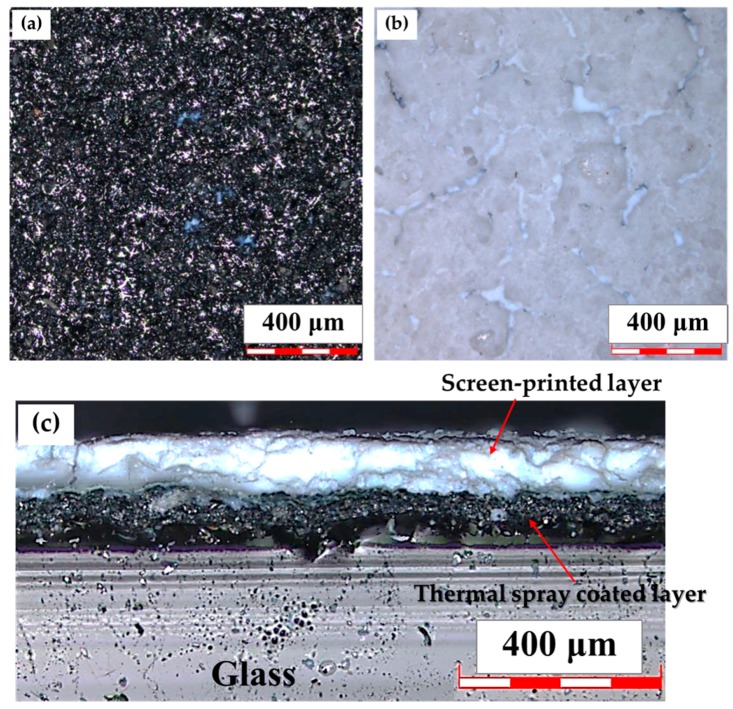
Optical images obtained by confocal microscope showing the (**a**) top view of thermal spray coated film; (**b**) top view of screen-printed film; and (**c**) cross section of the screen-printed layer on the thermal spray coated layer.

**Figure 2:**
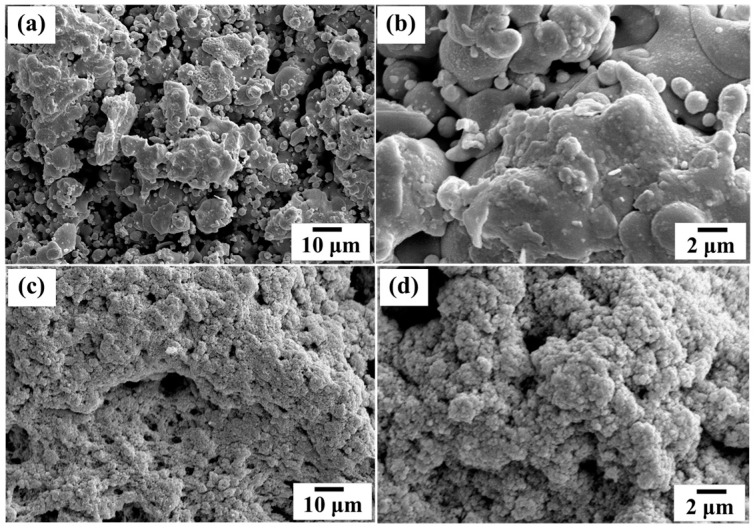
Field emission scanning electron microscopy (FESEM) images of the TiO_2_ film surface fabricated by (**a**,**b**) thermal spray coating and (**c**,**d**) screen-printing.

Figure 1a,b show the top surface of the coated films obtained by thermal spray coating and screen printing, and the cross section of those films is shown in Figure 1c. The film thickness achieved by the thermal spray coating and the screen printing is approximately 50 μm and 100 μm, respectively, with the color of the films appearing in dark gray and white. The coated layer was found perfectly adherent to its support—the thermal spray layer on the sand blasted glass surface and the screen-printed film on the thermal spray coated layer. Adhesion of the screen-printed films appeared to improve due to the large groove dimension and a certain degree of surface roughness on the thermal spray layer. Particularly for the screen-printed film, the TiO_2_ paste formulation and multi-layered film fabrication needed to be developed and seamlessly interfaced towards the improvement of the film adhesion. The mixture of viscous binder and the right amount of TiO_2_ powder could help regulate the paste viscosity while allowing for the increased porosity to be created in the sintered film, with the pore width of 99–156 Å found to be the most critical film property in terms of mechanical stability and adhesion enhancement to the substrate [36]. The binder is used to create voids between the particles and acts as an important role during sintering as it subsequently affects the film density, the topological structure, and the final strength of the film. A proper film thickness and film stability are strongly required regarding the environmental concern to the release of TiO_2_ during the wastewater treatment [37,38]. Top view topology of the entire screen-printed film revealed a uniform dispersion of particle agglomerates (see Figure 2b).

**Figure 3:**
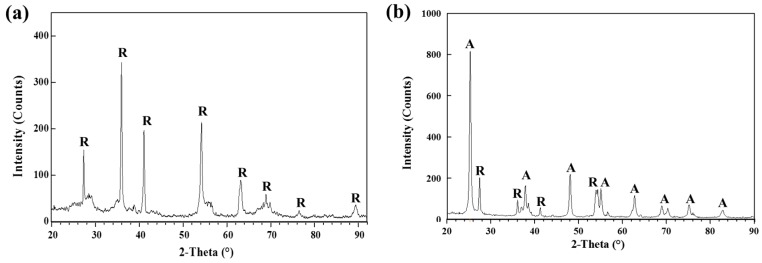
X-ray diffraction patterns of the films fabricated by (**a**) thermal spray coating and (**b**) screen printing. The marked letters of A and R indicate respectively the TiO_2_ phases of anatase and rutile.

The crystallographic phase of TiO_2_ coated films by thermal spray coating and screen printing was characterized by X-ray diffraction (XRD) (see Figure 3a,b). The crystallite size of TiO_2_ particles on the fabricated films was calculated by the Scherrer equation through full width at half maximum (FWHM) of the diffraction peaks of anatase (101) and rutile (110) [39]. The thermal spray coated film resulted solely in the rutile phase, with the calculated crystallite size of 79 nm. However, the screen-printed film has shown the mixed phase of anatase and rutile in a crystalline structure ratio of 80:20, with the calculated crystallite size of 20 nm and 27 nm, respectively.

**Figure 4:**
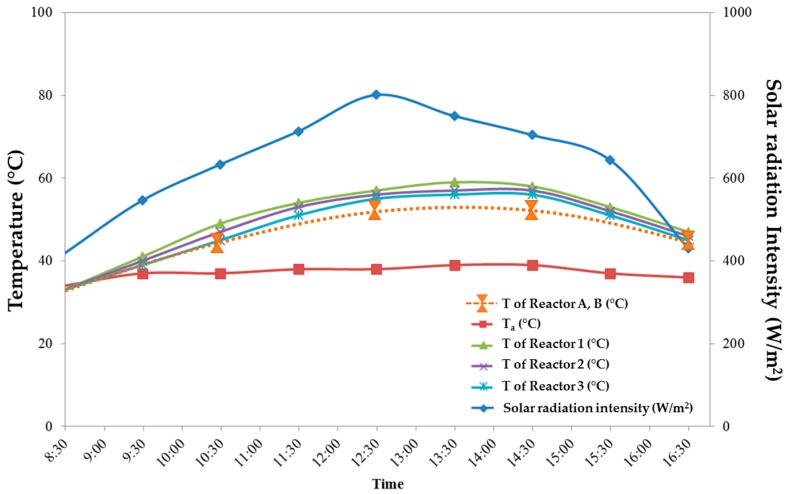
Plots of solar radiation intensity, ambient air temperature (T_a_), and reactor temperature during operation time from 08:30 a.m.–4:30 p.m.

After assembling the glasses with the fabricated films into the reactors, the experiment was performed. Solar radiation intensity and temperatures of all reactors were monitored over the entire period of operation; the results are shown in Figure 4. During the operation, the temperature of water samples in all reactors increased from about 35 °C to its maximum in a window range of 20 °C. The maximum temperature is 53 °C for Reactors A and B, and, respectively, 59, 57, 56 °C for Reactors 1, 2, and 3 after 6 h contact time. The temperature of the inactivation reactors appeared to be in parallel dependent upon the intensity of solar radiation. The maximum solar radiation intensity was found at 12:30 p.m.; 801 W/m^2^.

Performance of the TiO_2_ fabricated film on *E. coli* inactivation was investigated as a function of contact time. In this study, the experiment was designed to evaluate the bactericidal activity of the TiO_2_ films assembled reactors and compare their effectiveness in view of different light sources and ratios of the fabricated film area per volume of water on the cells inactivation. The total numbers of living *E. coli* cells collected at each operation time interval are shown in Table 1; the corresponding results in percentage numbers of living *E. coli* cell counts over the 8 h of operation are shown in Table 2.

**Figure 5:**
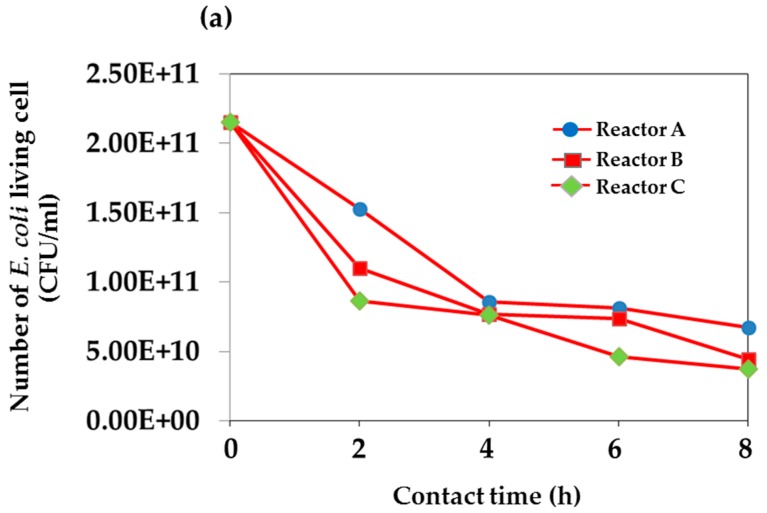

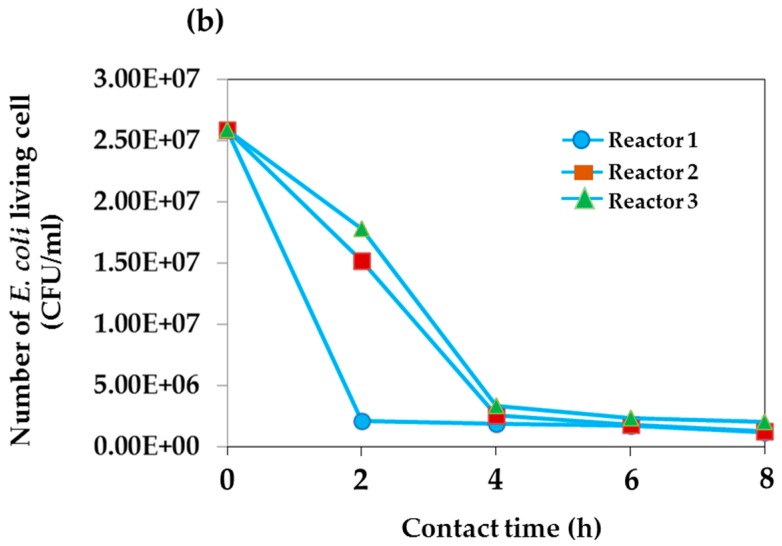
Plots of total number of *E. coli* living cells (CFU/mL) and contact time (h) obtained from the test of reactor under exposure to sunlight over 8 h operation period for (**a**) Reactors A, B, and C; and (**b**) Reactors 1, 2, and 3.

Figure 5a shows the plots of total numbers of *E. coli* living cells against contact time for Reactors A, B, and C. Using the *E. coli* initial concentration of 2.15 × 10^11^ CFU/mL, the effect of UV light for Reactor C has undoubtedly shown its more significant effect on the inactivation of the *E. coli* cells than that of the sunlight, Reactor B. The percentage reduction of *E. coli* determined for the UV light and the sunlight conditions are, respectively, 59.8% and 48.8%. The explanation regarding the photocatalytic inactivation ability of TiO_2_ has been described in many reports [40,41,42]. With relatively low energy of UV light, the cells could be damaged through the oxidation stress caused by the oxygen radicals within the cells [43]. The reactor without the fabricated TiO_2_ film showed the lowest percentage reduction, 28.8%, of *E. coli* after the 2 h contact time.

The initial concentration was adjusted to the range of 2.59 × 10^7^ CFU/mL and the dilution effect was found to enhance the *E. coli* inactivation ability. Figure 5b demonstrates the plots of total numbers of *E. coli* living cells against time as a function of water volume used in Reactors 1, 2, and 3; i.e., 660 cm^3^, 1320 cm^3^, and 1980 cm^3^, respectively. At the beginning of the experiment, the initial quantity of total living *E. coli* in Reactors 1–3 was fixed at the same concentration of 2.59 × 10^7^ CFU/mL or 100%. The curve for Reactor 1 appeared to drastically decrease within the first 2 h, compared to the gradual decrease over the 4 h contact time for Reactors 2 and 3. For a given condition employed in this study, the percentage of numbers of living *E. coli* cells was found to be 8.1% for the area of screen-printed TiO_2_ film:volume of water in the reactor was 1:2.62. After increasing the water volume in the ratio by a factor of 2 or 3, the *E. coli* reduction efficiency did not improve, as seen for Reactors 2 and 3. After 4 h, all curves slowly declined until the end of the testing period. For the first 2 h of contact time, the surface area of photocatalytic active species is believed to play a predominant role affecting the cells’ inactivation, as water volume is relatively smaller. As a consequence, there would be an increasing degree of adsorption and hence inactivation ability due to the greater availability of bacteria on the photocatalyst surface [17]. In this case, the combined effect of water temperature in the reactors of approximately less than 50 °C was considered to be insignificant. During this first operation period, the temperature of water sample in Reactors 1–3 was less than 50 °C. It was particularly noted in the literature that the water temperature up to 45 °C has no effect on the inactivation of *E. coli*, as the temperature higher than 50 °C due to radiation could promote the pasteurizing effect on the bacteria cells [44].

**Figure 6:**
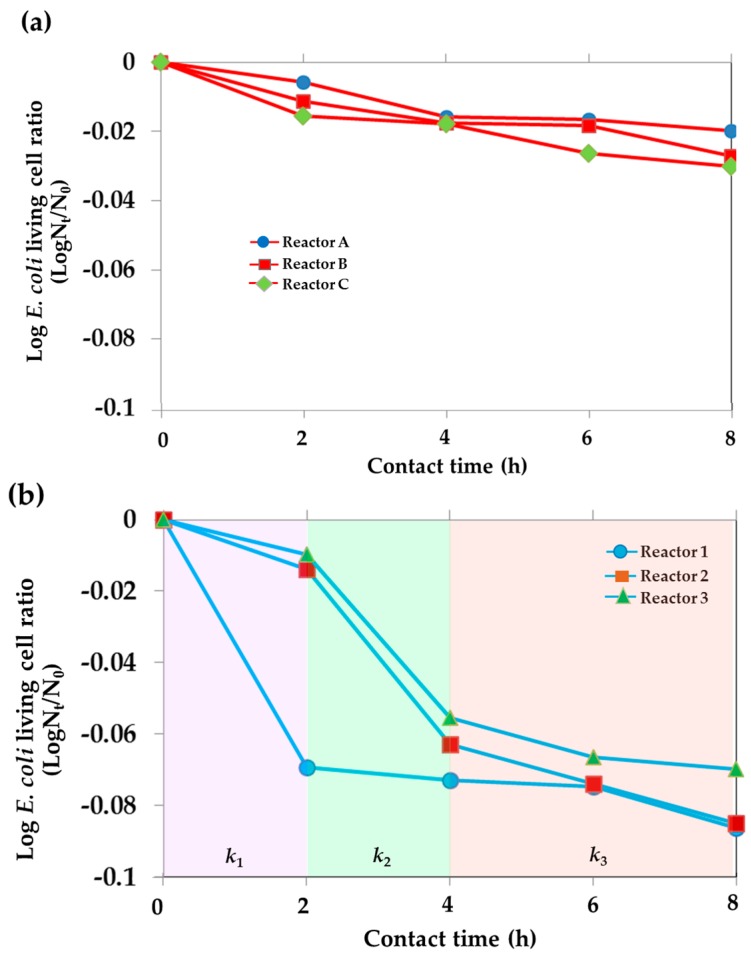
Kinetic data plots of *E. coli* inactivation for (**a**) Reactors A, B, and C and (**b**) Reactors 1, 2, and 3.

In this study, the efficiency of the reactors on reducing the *E. coli* cells as a function of contact time was evaluated in terms of kinetic rate, as the plots shown in Figure 6. Figure 6a appears to show a linear dependence of *E. coli* inactivation ability upon contact time over the 8 h duration. For Reactors A, B, and C, the inactivation follows the first order dependence (Log_10_(N_t_/N_0_) = −*k*t, where N_t_ = total number of *E. coli* living cells at time t, N_0_ = total number of *E. coli* living cells at time 0, and *k* = rate constant). As presented in Table 3, it is apparent that *k_Reactor C_* > *k_Reactor B_* > *k_Reactor A_*, confirming the photocatalytic inactivation of the TiO_2_ film that is considerably sensitive to UV during 8 h of operation.

The curves in Figure 6b for Reactors 1, 2, and 3 also show their behavior in time dependence of cell inactivation, but not in such linear behavior; thus, different *k* values were examined. During 0–2 h, the rate *k*_1_ of Reactor 1 is higher than that of Reactors 2 and 3; i.e., 0.0691, 0.0138, and 0.0096. Thereafter, from 2 h to 8 h, the behavior of the rest of the number of living *E. coli* cells in the reactor declining directly with the operation time follows the first order dependence. The inactivation ability was found to decrease to about ten times lower; the rate *k*_2_ is 0.0053. Reducing the initial concentration of *E. coli* by four log_10_ stages in water samples, the inactivation performance of Reactor 1 has improved about ten times higher for the rate of Reactor 1 with respect to Reactor B, under exposure to sunlight. It could be presumed that the diluted concentration of *E. coli* cells in the water sample has become a facilitating effect on inhibiting the *E. coli* number.

On further increase in water volume, in turn, the slower rate of *E. coli* inactivation is obtained. For Reactors 2 and 3, three different rates of inactivation are clearly observed. The rate *k*_2_ is largest during 2–4 h of operation, probably due to the greater adsorption of reaction partners to the TiO_2_ surface, leading to increased effectiveness of the catalysis and thus the enhancement of cell inactivation [45]. During 0–2 h, the adsorption is probably limited by the key factor of water volume determining the mobility of the reaction species to the catalytic surface. For the 2–4 h period, a certain amount of the cells adsorbing on the surface could possibly alter the surface property of the catalytic film. That is, the total surface charge of TiO_2_ has been altered, to some extent, to become more hydrophobic [46]. As a result, such phenomena could probably induce more adsorption of different charge from water medium, subsequently establishing a certain mass movement of water towards the adsorbate. This hereby could lead to more adsorption of the bacteria on the surface as dependent upon the contact time. The mechanism of mass movement inducing adsorption due to the alternation of surface charge is believed to be an important factor facilitating the cells’ inactivation ability, whereas the reaction rate *k*_3_ is prone to linear behavior, as the cells inactivation ability also depends on the concentration of living cells left after largely being inhibited in the first four hours.

## 3. Materials and Methods

### 3.1. Film Preparation and Characterization

**Figure 7:**
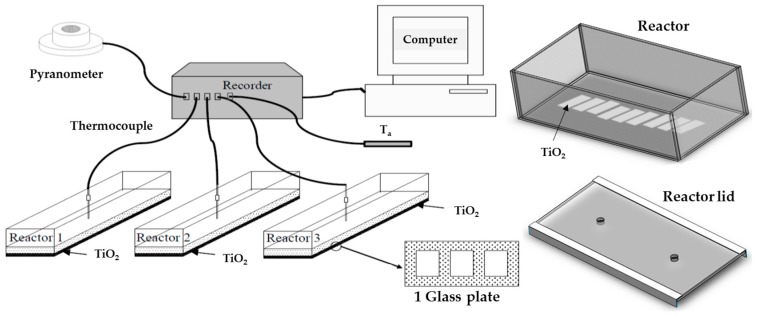
Schematic drawing illustrating connections of the experimental devices employed in the study of inactivation of bacteria.

The layered TiO_2_ films were prepared on glass plates 10 cm × 15 cm in size and were cleaned with soap, rinsed with iso-propanol, and dried in an oven. The entire surface of the glass plates was prepared by sand blasting and thermal spray coated with TiO_2_ (Amperit^®^ Flame and Plasma Spray Powders, H. C. Starck GmbH, Munich, Germany) and plasma spray powder No. 782.2, 95% of 90 μm size range). The feed rate of thermal spray coating was held fixed at 53 g/min in H_2_ and Ar air flow, and the thickness was controlled at 50 μm. The second layer of TiO_2_ film was casted by a screen printing technique on top of the thermal spray coated film. The TiO_2_ paste was prepared by mixing 20% wt TiO_2_ (P25 Degussa, Evonik, Hanau, Germany) with 5% Poly (vinyl alcohol) (PVA) in de-ionized water, and coated on the marked area on the thermal spray coated film. In each glass plate, there are three sectional areas of 8 × 3.5 cm^2^ for screen printing (see the drawing of 1 glass plate in Figure 7). The total area of the screen-printed film per glass plate is 84 cm^2^. For each glass plate, five replicate layers were done in order to achieve thickness of about 100 μm, as each layer of screen printing was subject to oven drying at 100 °C for 1 h prior to applying its new layer. The annealing process of the screen-printed films was done at 350 °C for 3 h with the ramping rate of 1 °C/min. The cross-sectional view of the TiO_2_ thermal spray coated film was characterized by confocal microscope (Confocal Olympus OLS 400, Tokyo, Japan) as the thickness was also measured. The phase of the TiO_2_ screen-printed film was identified by X-ray diffraction (X’Pert PRO PANalytical, PANalytical BV, Almelo, The Netherlands).

### 3.2. Inactivation Reactor

The inactivation reactor was designed to be composed of a base and a cover. The base was made of a box of 3 mm glass in a volume of 15 × 30 × 10 cm^3^, inserted with three glass plates into the inactivation unit; thus, the total area of the TiO_2_ screen-printed film per reactor is 252 cm^2^. The lid of the reactor is a transparent glass sheet, with two holes pierced for putting thermocouples and collecting water from the reactor. The experiment was conducted under sunlight, as intensity of solar radiation was measured by pyranometer (KIPP & ZONEN, CM11, Delft, The Netherlands) and recorded through a Wisco’s Analog Input Module (model AI210, Bangkok, Thailand), while the temperature was also recorded simultaneously. The schematic diagram of the experimental device is illustrated in Figure 7. In this study, there were 6 types of reactors set in different conditions to obtain a better insight into the efficiency of each reactor under various fabrication/test conditions on the bactericidal ability. The conditions for Reactors A, B, C, 1, 2, and 3 are as follows: Reactor A—660 cm^3^ water volume with *E. coli* and exposed to sunlight; Reactor B—660 cm^3^ water volume with *E. coli*, 0.0252 m^2^ area of screen-printed TiO_2_ with 1:2.62 of TiO_2_ film:water volume ratio, and exposed to sunlight; Reactor C—660 cm^3^ water volume with *E. coli*, 0.0252 m^2^ area of screen-printed TiO_2_ with 1:2.62 of TiO_2_ film:water volume ratio, and exposed to UV light; Reactor 1—660 cm^3^ water volume with *E. coli* and 1:2.62 of TiO_2_ film:water volume ratio, and exposed to sunlight; Reactor 2—1320 cm^3^ water volume with *E. coli* and 1:5.24 of TiO_2_ film:water volume ratio, and exposed to sunlight; Reactor 3—1980 cm^3^ water volume with *E. coli* and 1:7.86 of TiO_2_ film:water volume ratio, and exposed to sunlight.

### 3.3. Unit Testing

The unit was tested under sunlight and UV light for a set period of time; the operation period started from 08:30 a.m. to 16:30 p.m. UV light source was a 125 W lamp with a 0.52 filter. The experiment was designed to study the inactivation efficacy of the reactor, with no flow of water, as the volume of water was varied; 660 cm^3^, 1320 cm^3^, and 1980 cm^3^ designated for Reactor 1, Reactor 2, and Reactor 3, respectively. A tap water sample was boiled and cooled down to room temperature. *Escherichia coli* (ATCC^®^ 25922™) was cultured in Nutrient Broth (NB) for 24 h and stored in the refrigerator at 4 °C. The required *E. coli* was adjusted by dilution. The initial concentration was prepared at 10^7^ and 10^11^ CFU/mL.

### 3.4. Sample Collection

Water from the reactors was sampled at two-hour intervals during the operation for counting the total number of *E. coli* living cells; 10 mL of water was collected into sterilized tubes. The samples were then tested with eosin methylene blue (EMB) by incubating at 30 °C for 24 h; and the total number of *E. coli* living cells was counted. The percentage reduction of *E. coli* was calculated through the following expression; % reduction = (a − b) × 100/a, where a is number of viable cells (CFU/mL) in the control, and b is number of viable cells (CFU/mL) in the sample.

## 4. Conclusions

The efficiently photocatalytic performance of layered TiO_2_ films on treating *E. coli* cells was investigated. The double-layered TiO_2_ films with good adhesion were successfully produced by thermal spray coating and screen printing. The film fabrication was manipulated to achieve the total film thickness of approximately 150 µm. The crystalline structure ratio of anatase:rutile for the screen-printed film was characterized as 80:20, as the calculated crystallite size of rutile is larger than that of anatase. The experiment was conducted using two sets of initial *E. coli* concentrations of 2.15 × 10^11^ and 2.59 × 10^7^ CFU/mL. The optimized film area:water volume of 1:2.62 has shown a significant ability to reduce the *E. coli* cells in water samples to 8.1%, under the exposure to sunlight and a given condition employed in this study. The kinetic response of the reactors to the *E. coli* cells was found to be a linear dependence upon the contact time for the reactors under exposure to UV and sunlight. The mechanism of mass movement inducing adsorption due to the alternation of surface charge was proposed as an important factor facilitating the cells inactivation ability. This work has shown the use of TiO_2_ P25 as a catalyst in a merit of a design fabrication of low-cost, layered TiO_2_ film assembled reactors with their potential in *E. coli* cell inactivation. To some extent, this aspect of work could pave the way towards a consideration of scalable reactors as a possibly complementary treatment process.

## Figures and Tables

**Table 1:** Total numbers of *E. coli* living cells in the reactors at each operation time interval.

Time	Total Number of Living *E. coil* (CFU/mL)					
	Reactor A ^1^ (Water + *E. coli* + Sun)	Reactor B ^1,†^ (Water + *E. coli* + TiO_2_ + Sun)	Reactor C ^1,†^ (Water + *E. coli* + TiO_2_ + UV)	Reactor 1 ^†^	Reactor 2 ^†^	Reactor 3 ^†^
8:30	2.15 × 10^11^	2.15 × 10^11^	2.15 × 10^11^	2.59 × 10^7^	2.59 × 10^7^	2.59 × 10^7^
10:30	1.53 × 10^11^	1.10 × 10^11^	8.65 × 10^10^	2.10 × 10^6^	1.52 × 10^7^	1.78 × 10^7^
12:30	8.55 × 10^10^	7.70 × 10^10^	7.60 × 10^10^	1.85 × 10^6^	2.60 × 10^6^	3.35 × 10^6^
14:30	8.15 × 10^10^	7.40 × 10^10^	4.65 × 10^10^	1.75 × 10^6^	1.80 × 10^6^	2.30 × 10^6^
16:30	6.75 × 10^10^	4.45 × 10^10^	3.75 × 10^10^	1.20 × 10^6^	1.25 × 10^6^	2.05 × 10^6^

**^1^** Volume of water in the reactor is 660 cm^3^; **^†^** Area of screen-printed TiO_2_ film is 0.0252 m^2^. The ratio of TiO_2_ film:water volume in Reactors B and C is 1:2.62. Volume of water in Reactors 1, 2, and 3 is 660, 1320, and 1980 cm^3^; thus, the ratio of TiO_2_ film:water volume in the reactor is 1:2.62, 1:5.24, 1:7.86, respectively in that order.

**Table 2:** Percentage of number of *E. coli* living cells in the reactors at each operation time interval.

Contact Time (h)	Percentage of Number of Living *E. coli* (%)					
	Reactor A ^1^ (Water + *E. coli* + Sun)	Reactor B ^1,†^ (Water + *E. coli* + TiO_2_ + Sun)	Reactor C ^1,†^ (Water + *E. coli* + TiO_2_ + UV)	Reactor 1 ^†^	Reactor 2 ^†^	Reactor 3 ^†^
0	100	100	100	100	100	100
2	71.2	51.2	40.2	8.1	58.7	68.7
4	39.8	35.8	35.4	7.1	10.0	12.9
6	37.9	34.4	21.6	6.8	6.9	8.9
8	31.4	20.7	17.4	4.6	4.8	7.9

**Table 3:** Rate constant determined from slope of the plots in Figure 6a,b at each operation period.

Condition	Rate Constant (*k*)_[Contact Time Duration]_		
Reactor A (Water + *E. coli* + Sun)	0.0050_[2–8 h]_		
Reactor B (Water + *E. coli* + TiO_2_ + Sun)	0.0061_[2–8 h]_		
Reactor C (Water + *E. coli* + TiO_2_ + UV)	0.0071_[2–8 h]_		
	*k*_1[0–2 h]_	*k*_2[2–4 h]_	*k*_3[4–8 h]_
Reactor 1 (TiO_2_ film area:water volume = 1:2.62)	0.0691	0.0053_[2–8 h]_	
Reactor 2 (TiO_2_ film area:water volume = 1:5.24)	0.0138	0.0490	0.0110
Reactor 3 (TiO_2_ film area:water volume = 1:7.86)	0.0096	0.0458	0.0072
